# The association between serum albumin and depression in chronic liver disease may differ by liver histology

**DOI:** 10.1186/s12888-021-03647-8

**Published:** 2022-01-04

**Authors:** Junyan Cao, Weihong Qiu, Yong Yu, Na Li, Huixiang Wu, Zhaocong Chen

**Affiliations:** 1 Department of Medical Ultrasonics, The Third Affiliated Hospital of Sun Yat-sen University, Sun Yat-sen University, 600 Tianhe Road, Guangzhou, 510630 China; 2 Department of Rehabilitation Medicine, The Third Affiliated Hospital of Sun Yat-sen University, Sun Yat-sen University, 600 Tianhe Road, Guangzhou, 510630 China

**Keywords:** Serum albumin, Depression, Chronic liver disease, Liver histology, Vibration controlled transient elastography

## Abstract

**Background:**

There are conflicting results regarding the association between chronic liver disease (CLD) and depression and the underlying biological mechanisms are lack of investigation. To address the impact of depression and its effects on the management of CLD, its biological marker is critical to be identified. The present study explored the association between serum albumin and depression in CLD patients and whether the association varied in different liver histological stages.

**Methods:**

Based on the United States National Health and Nutrition Examination Survey 2017–2018, the data of serum albumin and depressive symptoms from 627 participants with CLD were used. Depression symptoms were assessed with the nine-item Patient Health Questionnaire (PHQ-9). We used multivariate linear regression to evaluate the association between serum albumin and PHQ-9 scores. Stratified analysis was performed according to the liver histology examined by vibration controlled transient elastography.

**Results:**

Serum albumin level was inversely associated with PHQ-9 scores in the multivariate regression model after adjusting for mainly potential confounders (β = − 1.113, 95% CI: − 2.065 to − 0.162, *P* = 0.0221). In the subgroup analysis stratified by gender, controlled attenuation parameter (CAP) and liver stiffness measurement (LSM), the inverse association remained significant in female (β = − 2.002, 95% CI: − 3.515 to − 0.489, *P* = 0.0100), patients with CAP < 274 dB/m (β = − 2.215, 95% CI: − 3.621 to − 0.808, *P* = 0.0023) and patients with LSM ≥8.2 kPa (β = − 4.074, 95% CI: − 6.237 to − 1.911, *P* = 0.0003). Moreover, the association was much stronger when the serum albumin was higher than 3.4 g/dL among patients with LSM ≥8.2 kPa (β = − 4.835, 95% CI: − 7.137 to − 2.533, *P* < 0.0001).

**Conclusion:**

Our study revealed an inverse association between serum albumin and depression in CLD patients and this association differed according to liver histological changes. Serum albumin could be a warning marker for depressive symptoms in CLD patients. It is essential for taking corresponding intervention strategies.

## Introduction

Chronic liver disease (CLD) has been a serious economic burden to the society with a high prevalence and incidence [1]. It was estimated that at least 844 million people worldwide suffered from CLD and there were 2 million deaths per year [2]. In addition to progressive destruction of the liver parenchyma, multiple extrahepatic manifestations such as depression, fatigue, and compromised health-related quality of life were present in CLD [3, 4].

CLD has been associated with depression. A study showed that the prevalence of depression in chronic hepatitis B (CHB) and chronic hepatitis C (CHC) patients was significantly higher than healthy participants [5]. Likewise, it was reported that the percentage of CHB patients suffering from severe depression was higher than hepatitis B surface antigen carriers and healthy controls [6]. In another study utilizing National Inpatient Sample data, patients with alcohol-related liver disease (ALD) were 1.83 times more likely to have depression compared to those with chronic liver diseases not caused by alcohol [7]. Similar finding was found when they compared patients with ALD and those without liver diseases (OR 1.14). Another increasingly important cause of CLD is non-alcoholic fatty liver disease (NAFLD). Relatively high prevalence of depression has been identified in patients with NAFLD in several population-based studies [8–10]. However, most studies above focused on the association between depression and CLD, while the underlying biological mechanisms are lack of investigation.

Depression was expected to be the world’s third leading cause of disability according to the Global Burden of Disease 2017 [11]. In many patients with depression, their adherence to dietary intake and exercise is poor [9]. Moreover, depression is along with poor compliance with medication for general chronic illnesses [12]. To address the impact of depression and its effects on the management of CLD, the biological marker for concomitant depressive symptoms is critical to be identified and promptly intervened in patients with CLD.

Serum albumin is the most abundant protein in human blood plasma and is essential for the maintenance of pH, osmotic pressure and the transportation of fatty acids. Albumin level has been reported to be significantly lower in patients with major depressive disorders [13]. A study has demonstrated that low level of serum albumin is associated with long-term depressive symptoms in stroke surviving elderly individuals [14]. For the patients with schizophrenia, serum albumin level was significantly lower and decreased albumin level was associated with the depressive score of Positive and Negative Syndrome Scale [15]. Serum albumin level was also found to be closely associated with depressive symptoms in patients with maintenance hemodialysis [16].

To date, however, no study has examined the relationship between serum albumin level and depression in CLD patients. Previous studies have stated that depression tended to be associated with histological changes of liver diseases [10, 17]. Vibration controlled transient elastography (VCTE) is one of the most validated techniques suitable for assessing liver histology in large populations [18]. Therefore, we conceived to explore the associations between serum albumin and depression in CLD patients and whether this association differ by the liver histology, using the data of National Health and Nutrition Examination Survey (NHANES) from 2017 to 2018. If there is an independent association between serum albumin and depression, it may suggest that serum albumin is a useful biomarker in identifying CLD patients at risk of developing depressive symptoms.

## Materials and methods

### Study population

The NHANES was a cross-sectional population-based national survey that collected information of the health and nutrition in the USA in 2-year cycles. Researchers throughout the world can access to the survey data online. Details regarding the design and operation of the datasets were provided at www.cdc.gov/nchs/nhanes/. The survey was approved by the ethics review board of the National Center for Health Statistics and written informed consent was obtained from all participants or their guardians.

Five categories of CLD were included: CHB (positive hepatitis B virus surface antigen), CHC (positive hepatitis C virus RNA), chronic hepatitis E (CHE, positive hepatitis E virus IgM), NAFLD and ALD. Although hepatitis E virus infection is usually asymptomatic, it can progress to chronicity and fibrosis especially in individuals with underlying liver diseases or immunodeficiency [19]. Therefore, we have also included CHE in this study. NAFLD was defined by the absence of any other causes of CLD and presence of elevated liver enzymes (alanine aminotransferase (ALT) > 40 U/L or aspartate aminotransferase (AST) > 37 U/L in men and ALT or AST > 31 U/L in women). ALD was defined as a significant alcohol use of > 30 g/day for men and > 20 g/day for women over past 12 months and the presence of elevated liver enzymes.

Of all the 679 participants with CLD aged ≥ 18 (from 18 to 80 years) with all serum albumin and nine-item Patient Health Questionnaire (PHQ-9) data from NHANES 2017–2018, 627 participants (348 men and 279 women, 43.6 ± 16.2 years for men and 50.7 ± 16.8 years for women) remained for the final analysis after exclusion of 52 participants with cancer or malignancy.

### Variables

In this study, the independent variable was serum albumin. Its concentration was measured using the bichromatic digital endpoint method on a Roche Cobas 6000 (c501 module) analyzer. In the reaction, albumin bound with dye bromocresol purple to form a complex. The absorbance was tested at 600 nm. The secondary wavelength was 700 nm. The dependent variable was depressive symptom. It was assessed using the PHQ-9. Each of the nine items was scored by a 4-point response ranging from 0 to 3 and the sum-score was used as a severity measure.

NHANES provided information about age, gender, race, body mass index (BMI), alcohol consumption, serum total bilirubin (TB), ALT, AST, antidepressant use, controlled attenuation parameter (CAP) and liver stiffness measurement (LSM). Alcohol consumption was estimated according to self-reported amount and frequency of alcohol use in the previous year. Antidepressant use including selective serotonin reuptake inhibitors, tricyclics, monoamine oxidase inhibitors, tetracyclics, serotonin and norepinephrine reuptake inhibitors and miscellaneous antidepressants was determined through the in-home questionnaire. VCTE is a non-invasive tool that can be used to measure CAP and LSM, which is available for assessing the existence of liver steatosis and fibrosis, respectively. In the NHANES 2017–2018 cycle, VCTE was implemented on FibroScan® 502 Touch model (M Probe; XL Probe; Echosens, Paris, France). According to a recent study, liver steatosis was defined with CAP ≥274 dB/m, and liver fibrosis was defined with LSM ≥8.2 kPa [20]. Although liver steatosis was more common in NAFLD, it could also occur in chronic hepatitis [21, 22] and ALD [23]. According to published meta-analysis and prospective studies, the liver stiffness cut-off was 8.2 kPa for F ≥ 3 in CHB [24], whereas, in NAFLD, 8.2 kPa was the cut-off for F ≥ 2 [20]. NAFLD had the highest proportion in our study population. Therefore, we chose 8.2 kPa as the threshold of liver fibrosis, ensuring that the participants with LSM ≥8.2 kPa do have liver fibrosis using this relative high value. Because the CLD population of our study was relatively small and there was only a total of 134 participants with LSM ≥8.2 kPa, hence they were not divided with advanced fibrosis and cirrhosis.

### Statistical analysis

All estimates were calculated using NHANES sample weights. Categorical variables were described by frequency distributions. Continuous variables were described by means ± standard deviation. Weighted linear regression models (continuous variables) and weighted chi-square tests (categorical variables) were used to calculate differences between different groups categorized by quintile of serum albumin. *P* < 0.05 was considered statistically significant. Multivariate linear regression models were used to evaluate the association between serum albumin and PHQ-9 scores in CLD patients. Age, gender and race were adjusted for the first model. The second model was further adjusted for BMI, alcohol consumption, TB, ALT, AST, antidepressant use, CAP and LSM. These covariates were selected either based on literature knowledge or if they changed the exposure coefficient by more than 10%. The category of CLD was not included as a covariate because it didn’t changed the exposure coefficient more than 10%. All statistical analyses were performed with R (http://www.R-project.org, The R Foundation) and EmpowerStats software (http://www.empowerstats.com, X&Y Solutions, Inc., Boston, MA).

## Results

Among the 627 participants included in our analysis, 21 (3.35%) were participants with CHB, 41 (6.54%) were participants with CHC, 55 (8.77%) were participants with CHE, 259 (41.31%) were participants with NAFLD and 251 (40.03%) were participants with ALD. The weighted sociodemographic and medical characteristics of the participants subclassified based on serum albumin quintiles were shown in Table 1. There were significant differences in baseline characteristics between the serum albumin quintiles including age, gender, BMI, alcohol consumption, CAP, LSM, TB, PHQ-9 scores and antidepressant use. It suggested that the values or the distributions were not the same for the quintiles of serum albumin and these variables should be considered as covariates while testing the trend between quintiles of serum albumin.

**Table 1:** The characteristics of participants

Serum albumin	All (*n*=627)	Q1 (*n*=86)	Q2 (*n*=110)	Q3 (*n*=135)	Q4 (*n*=154)	Q5 (*n*=142)	*P*-value
Age (years)	44.03 ± 16.31	48.56 ± 15.63	46.98 ± 16.27	47.67 ± 16.00	42.14 ± 16.64	39.84 ± 15.14	<0.0001
Gender (%)							<0.0001
Male	58.12	28.40	37.00	61.84	68.25	70.74	
Female	41.88	71.60	63.00	38.16	31.75	29.26	
Race (%)							0.5458
White	60.03	55.08	55.75	63.56	60.62	61.68	
Black	8.58	13.97	11.00	8.51	7.53	5.90	
Hispanic	20.04	19.54	25.65	18.98	17.84	20.01	
Other	11.36	11.41	7.60	8.94	14.00	12.42	
BMI (kg/m^2^)	31.89 ± 7.91	35.24 ± 11.22	34.40 ± 8.89	32.29 ± 7.66	31.66 ± 5.92	29.01 ± 6.11	<0.0001
Alcohol consumption (%)							0.0040
Never	7.21	6.47	8.69	6.09	4.08	10.45	
Mild	38.20	44.12	28.16	37.13	48.75	31.69	
Moderate	42.48	33.61	57.39	42.39	37.40	43.28	
Excessive	12.01	15.80	5.75	14.40	9.77	14.58	
CAP (%)							0.0029
<274 dB/m	38.42	51.23	24.70	33.99	37.54	43.86	
≥274 dB/m	61.58	48.77	75.30	66.01	62.46	56.14	
LSM (%)							0.0290
<8.2 kPa	79.96	68.71	77.30	78.01	82.13	85.60	
≥8.2 kPa	20.04	31.29	22.70	21.99	17.87	14.40	
ALT (U/L)	51.93 ± 29.72	47.52 ± 24.97	48.59 ± 29.16	53.61 ± 36.10	53.63 ± 29.46	52.98 ± 27.16	0.4032
AST (U/L)	40.79 ± 25.06	44.79 ± 31.25	40.41 ± 24.68	39.55 ± 23.56	37.86 ± 18.67	42.80 ± 27.86	0.2255
TB (mg/dL)	0.52 ± 0.29	0.38 ± 0.20	0.43 ± 0.23	0.54 ± 0.26	0.53 ± 0.31	0.62 ± 0.30	<0.0001
PHQ-9 score	3.25 ± 3.86	4.42 ± 4.45	3.61 ± 3.96	3.88 ± 4.56	2.76 ± 3.58	2.58 ± 2.98	0.0008
Antidepressant use (%)							0.0103
+	11.13	17.70	16.69	14.09	7.97	6.27	
-	88.87	82.30	83.31	85.91	92.03	93.73	

Mean ± SD for continuous variables, *P*-value was calculated by weighted linear regression

% for categorical variables, *P*-value was calculated by weighted chi-square test

*BMI* body mass index, *TB* total bilirubin, *ALT* alanine aminotransferase, *AST* aspartate aminotransferase, *PHQ-9* nine-item Patient Health Questionnaire, *CAP* controlled attenuation parameter, *LSM* liver stiffness measurement

**Fig. 1:**
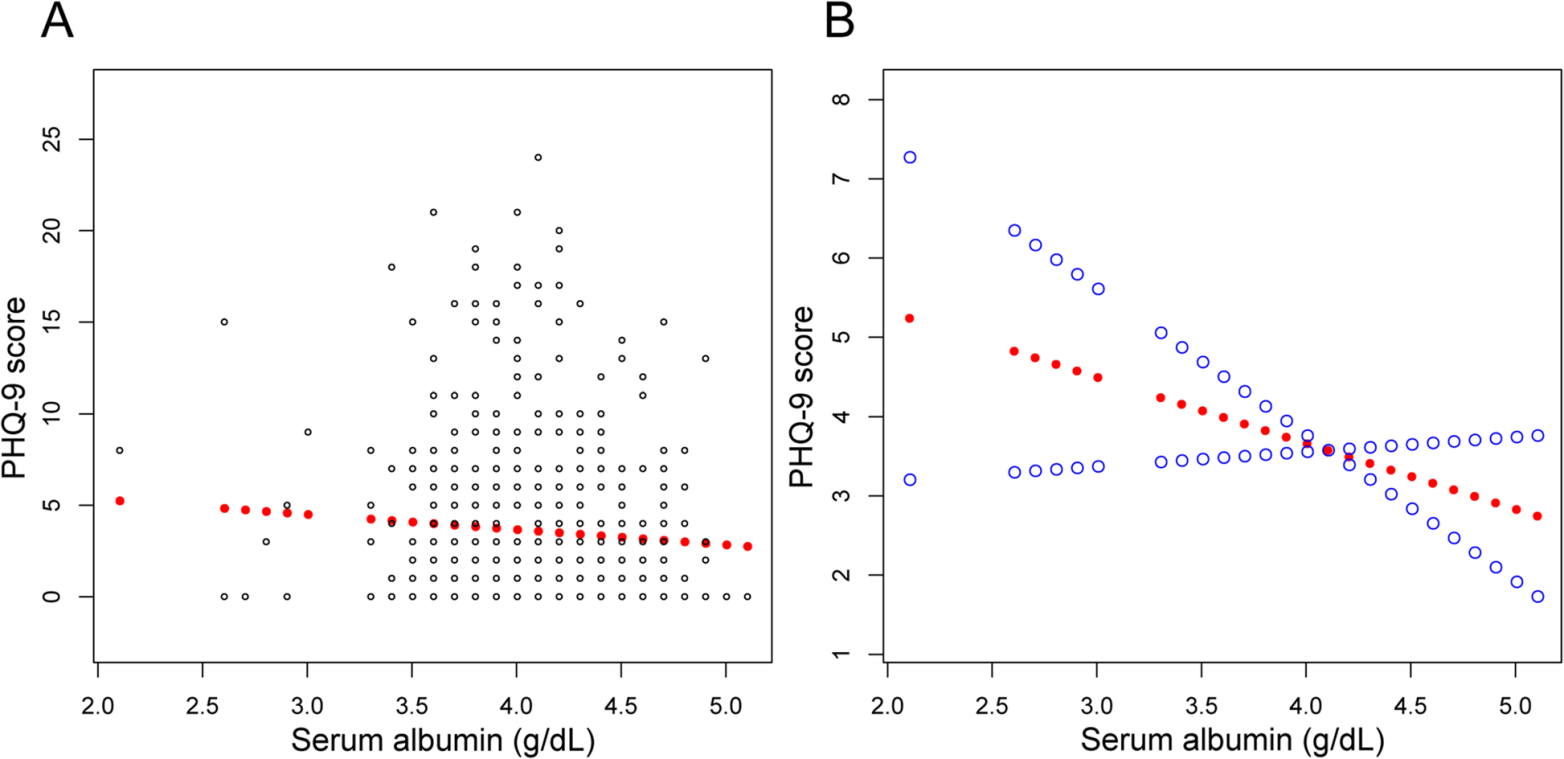
The association between serum albumin and PHQ-9 score. **A** Each black point represents a sample. **B** Solid rad line represents the smooth curve fit between variables. Blue bands represent the 95% of confidence interval from the fit. Age, gender, race, body mass index, alcohol consumption, total bilirubin, alanine aminotransferase, aspartate aminotransferase, antidepressant use, controlled attenuation parameter and liver stiffness measurement were adjusted

Three weighted univariate and multivariate linear regression models were constructed. In the unadjusted model, we observed an inverse association between serum albumin and PHQ-9 scores. Similar results were found in model 2 (adjustment for age, gender, race) (β = − 1.374, 95% CI: − 2.281 to − 0.466, *P* = 0.0031) and model 3 (fully adjusted model) (β = − 1.113, 95% CI: − 2.065 to − 0.162, *P* = 0.0221). Stratified by quintile of serum albumin, the trend test remained significant between them (*P* for trend = 0.009) (Table 2). Weighted generalized additive model and smooth curve fitting were also performed to evaluate the associations between them (Fig. 1).

**Table 2:** Association between serum albumin level (g/dL) and PHQ-9 score

	Unadjusted model β (95% CI)	Model 1 β (95% CI)	Model 2 β (95% CI)
Albumin	-1.776 (-2.628, -0.924)	-1.374 (-2.281, -0.466)	-1.113 (-2.065, -0.162)
*P*-value	<0.0001	0.0031	0.0221
Albumin (quintile)			
Q1	Reference	Reference	Reference
Q2	-0.812 (-1.957, 0.334)	-0.686 (-1.829, 0.457)	-0.960 (-2.084, 0.163)
Q3	-0.534 (-1.634, 0.566)	-0.203 (-1.322, 0.915)	-0.441 (-1.551, 0.670)
Q4	-1.659 (-2.687, -0.632)	-1.286 (-2.347, -0.226)	-1.281 (-2.353, -0.208)
Q5	-1.834 (-2.854, -0.815)	-1.414 (-2.475, -0.354)	-1.462 (-2.564, -0.359)
*P* for trend	<0.001	0.003	0.009

Unadjusted model: no covariates were adjusted

Model 1: age, gender and race were adjusted

Model 2: age, gender, race, body mass index, alcohol consumption, total bilirubin, alanine aminotransferase, aspartate aminotransferase, antidepressant use, controlled attenuation parameter and liver stiffness measurement were adjusted

**Fig. 2:**
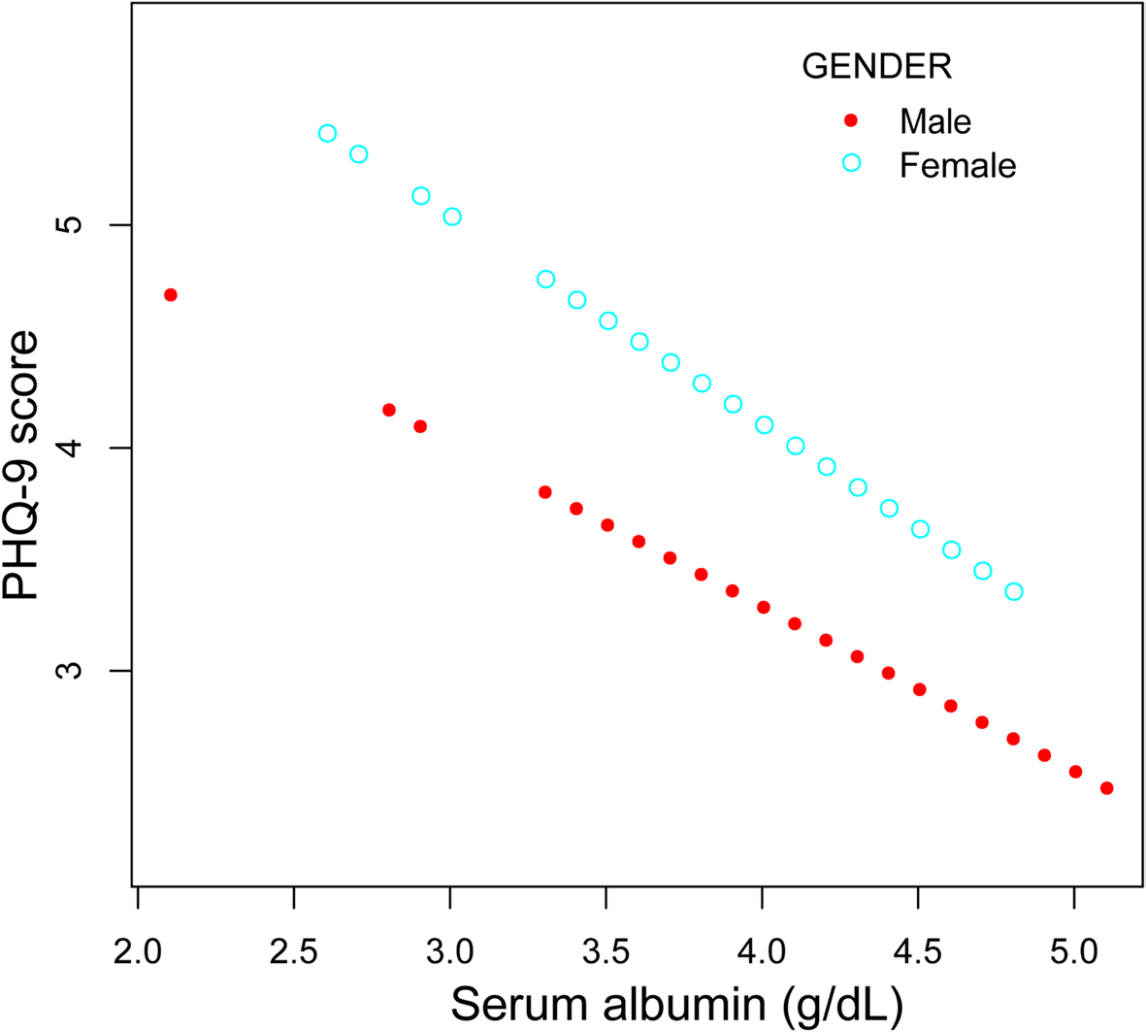
The association between serum albumin and PHQ-9 score, stratified by gender. Age, race, body mass index, alcohol consumption, total bilirubin, alanine aminotransferase, aspartate aminotransferase, antidepressant use, controlled attenuation parameter and liver stiffness measurement were adjusted

**Fig. 3:**
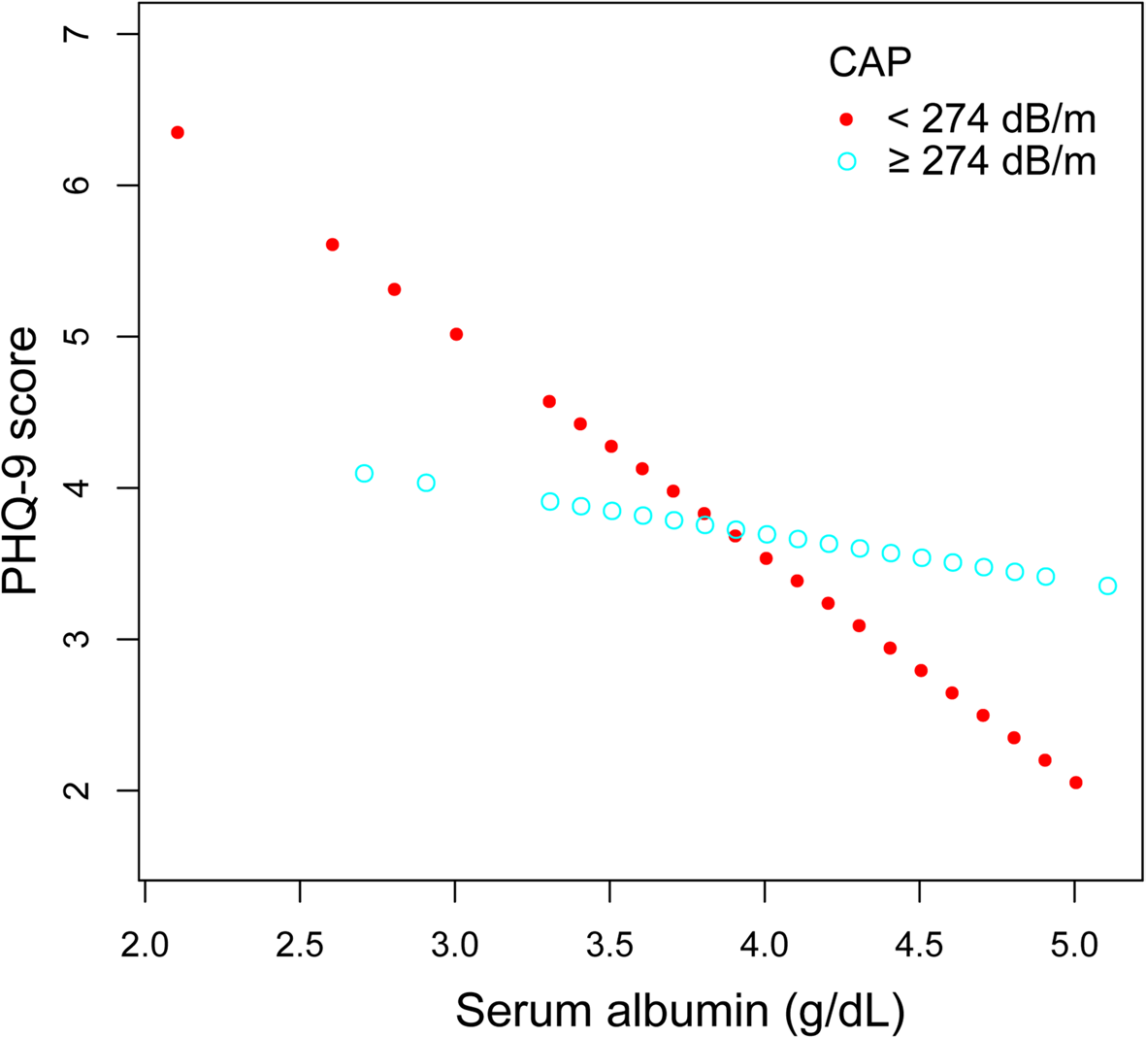
The association between serum albumin and PHQ-9 score, stratified by controlled attenuation parameter. Age, gender, race, body mass index, alcohol consumption, total bilirubin, alanine aminotransferase, aspartate aminotransferase, antidepressant use and liver stiffness measurement were adjusted

**Fig. 4:**
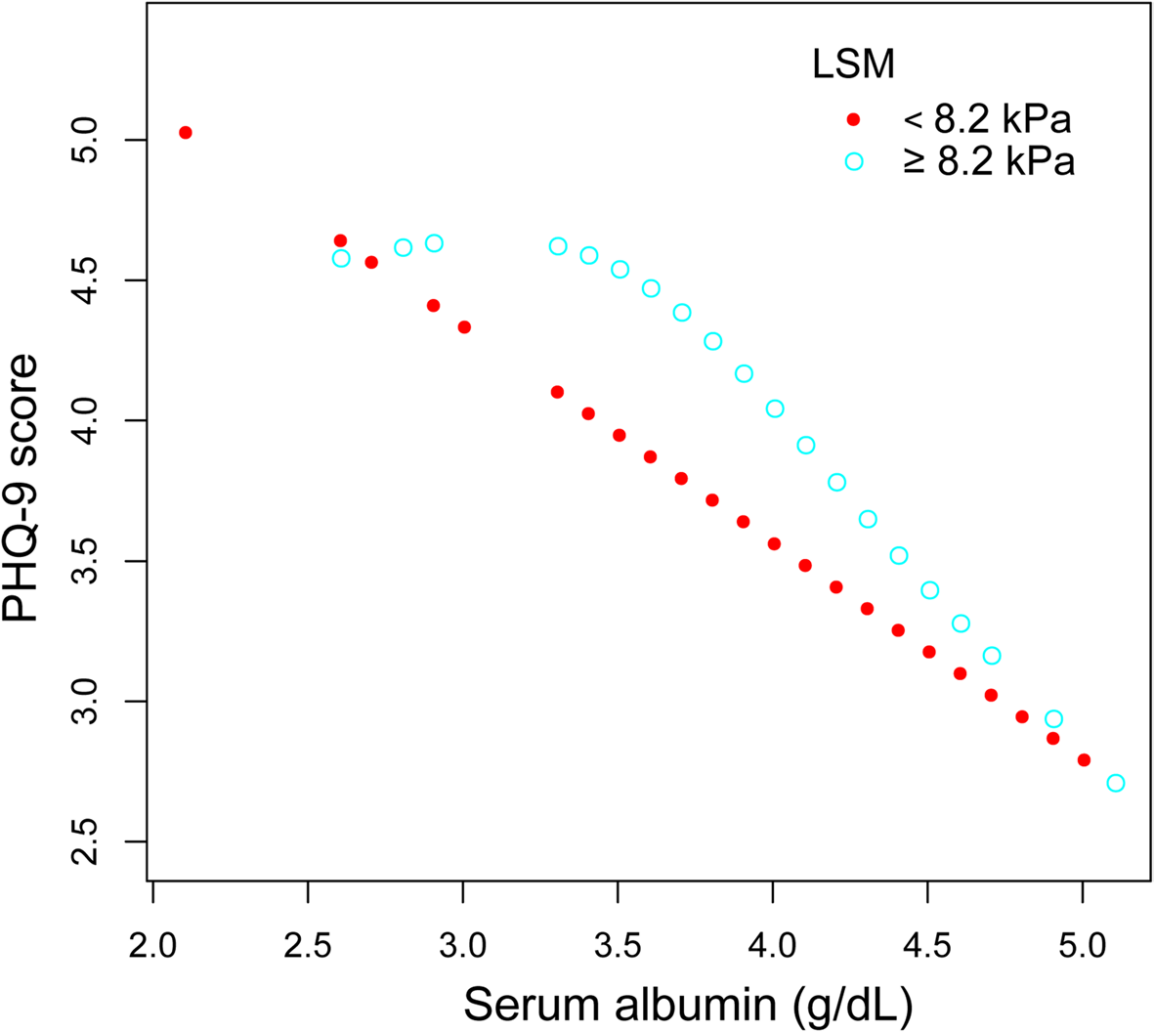
The association between serum albumin and PHQ-9 score, stratified by liver stiffness measurement. Age, gender, race, body mass index, alcohol consumption, total bilirubin, alanine aminotransferase, aspartate aminotransferase, antidepressant use and controlled attenuation parameter were adjusted

In the subgroup analysis (Table 3), serum albumin was significantly associated with lower PHQ-9 scores in female (β = − 2.002, 95% CI: − 3.515 to − 0.489, *P* = 0.0100), patients with CAP < 274 dB/m (β = − 2.215, 95% CI: − 3.621 to − 0.808, *P* = 0.0023) and patients with LSM ≥8.2 kPa (β = − 4.074, 95% CI: − 6.237 to − 1.911, *P* = 0.0003). Smooth curve fittings and generalized additive models used to characterize the relationship between serum albumin and PHQ-9 scores stratified by gender, CAP and LSM were shown in Figs. 2, 3 and 4. Among patients with LSM ≥8.2 kPa, there was a point of inflection at 3.4 g/dL of serum albumin identified using a two-piecewise linear regression model. The association was much stronger when the serum albumin was higher than 3.4 g/dL (β = − 4.835, 95% CI: − 7.137 to − 2.533, *P* < 0.0001).

**Table 3:** Association between serum albumin level (g/dL) and PHQ-9 score, stratified by gender, CAP and LSM

	Unadjusted model β (95% CI) *P*-value	Model 1 β (95% CI) *P*-value	Model 2 β (95% CI) *P*-value
Stratified by gender			
Male	-0.987 (-2.200, 0.227) 0.1119	-0.848 (-2.167, 0.470) 0.2082	-0.511 (-1.802, 0.779) 0.4380
Female	-1.824 (-3.135, -0.514) 0.0068	-2.033 (-3.371, -0.694) 0.0032	-2.002 (-3.515, -0.489) 0.0100
Stratified by CAP			
<274 dB/m	-2.491 (-3.684, -1.299) <0.0001	-1.809 (-3.098, -0.521) 0.0064	-2.215 (-3.621, -0.808) 0.0023
≥274 dB/m	-1.180 (-2.349, -0.011) 0.0485	-0.982 (-2.230, 0.266) 0.1238	-0.315 (-1.597, 0.968) 0.6309
Stratified by LSM			
<8.2 kPa	-1.556 (-2.524, -0.588) 0.0017	-1.075 (-2.108, -0.042) 0.0419	-0.320 (-1.381, 0.741) 0.5547
≥8.2 kPa	-2.453 (-4.325, -0.581) 0.0114	-2.294 (-4.334, -0.253) 0.0294	-4.074 (-6.237, -1.911) 0.0003

Unadjusted model: no covariates were adjusted

Model 1: age, gender and race were adjusted

Model 2: age, gender, race, body mass index, alcohol consumption, total bilirubin, alanine aminotransferase, aspartate aminotransferase, antidepressant use, controlled attenuation parameter and liver stiffness measurement were adjusted

In the subgroup analysis, the model is not adjusted for the stratification variable itself

*CAP* controlled attenuation parameter, *LSM* liver stiffness measurement

## Discussion

In this study, we used the representative samples of NHANES 2017–2018 to evaluate the association between serum albumin and depressive symptoms in CLD patients. Our results suggested that serum albumin was inversely associated with PHQ-9 scores in CLD patients. To be more specific, this association was significant in female, patients with CAP < 274 dB/m and patients with LSM ≥8.2 kPa.

Several studies have demonstrated conflicting results regarding the association between CLD and depression. Elwing et al. found that subjects with nonalcoholic steatohepatitis had significantly elevated PHQ-9 scores and increased lifetime rates of major depression disease [17]. Weinstein et al. reported increased prevalence of depression in patients with NAFLD and those with CHC [8]. In contrast, a study assessing the association between four types of CLD (CHB, CHC, ALD and NAFLD) with depression showed that depression was only strongly associated with CHC [25]. Additionally, a case-control study provided insignificant association between NAFLD and depression [26]. These conflicting data raised the possibility that there might be an unrevealing independent factor associated with depression in CLD patients, rather than CLD itself. In the present study, we found that serum albumin was inversely associated with depression in CLD patients. This finding was consistent with previous studies where the association between serum albumin and depression has been observed in elderly stroke survivors [14], patients with schizophrenia [15], HIV-infected persons [27] and individuals with a recent suicide attempt [28].

The etiology of depression is related to an excess of free-radicals, such as reactive oxygen and nitrogen species, which impose increased oxidative stress on the antioxidants defense system [29]. Abnormal levels of oxidative product have been found in peripheral blood [30], cerebrospinal [31] and postmortem brains [32] of the patients with depression. Aside from the property of protein binding and transportation, albumin is recognized to act as an antioxidant by scavenging free-radicals [33]. It has been found to be capable of binding iron and inhibiting lipid peroxidation [34]. These studies may indicate that it is through imbalance between lower antioxidants and increased oxidative stress that the protective antioxidant system fails and the decreased serum albumin influence the extent of depressive symptoms.

Although several biochemical indices such as US fatty liver index, hepatic steatosis index and fibrosis-4 index were reliable to assess the liver histological changes [35–37], they did not have an absolute cut-off and might misclassify the presence of steatosis or fibrosis. In comparison, VCTE is a new technology which is validated and feasible for large-scale examination and follow-ups [18]. The NHANES data from 2017 to 2018 adopted this kind of quantitative radiological information, producing more reliable estimates of both steatosis and fibrosis. This is the major strengths of our present study. To our knowledge, it remained rare to explore the association between serum albumin and depression and take the influence of liver histology into consideration.

Our subgroup analysis showed that the association between serum albumin and depression was not significant among patients with CAP ≥274 dB/m. As excess lipid accumulates in the cytoplasm, it could disrupt the cell constituent. The liver function is progressively compromised and various biochemical parameters, including alkaline phosphatase (ALP), AST, ALT and TB, would become abnormal. It has been reported that high bilirubin level was associated with depression [38]. Elevated ALT was found in adolescents with NAFLD who developed depression [39] and it was also a significant independent predictor for the occurrence of minor and major depression in adults [40]. Moreover, depression would escalate the histological changes in reverse, probably through the monoamine oxidase-A enzyme pathway [10]. These findings indicate that there might be more complicated mechanisms contributing to depression in liver steatosis. On the other hand, the category of CLD was not included in regression model as it did not satisfy the criteria of covariate selection, which could be partly due to relatively small sample size of each CLD category and its limited impact to exposure coefficient. We believed that there could be specific and complicated mechanisms underlying depression for each CLD category at the stage of liver steatosis. It is highly recommended to launch rigorous prospective cohort studies or molecular biological researches to address this question. Nevertheless, our result still make sense because it corroborate serum albumin as a potential early warning biomarker of depression in CLD patients.

Serum albumin is closely related with liver fibrosis and the albumin platelet product has been validated in liver fibrosis staging [41]. Furthermore, the functions of serum albumin, such as antioxidant and detoxification capacity and the ability to chelate metal ions, were compromised in patients with cirrhosis [42]. The dysfunction of binding toxic metabolites and inflammatory mediators potentially affects the systemic inflammation and antioxidant activities. For example, elevated free serum levels of inflammatory cytokines can affect the activation of indoleamine 2,3-dioxygenase and the degradation of tryptophan along the kynurenine pathway, and ultimately leads to reduction in the synthesis and availability of 5-hydroxytryptamine [43]. This process has been reported to be involved in the pathophysiology of major depressive disorder and suicide [44].

Meanwhile, serum albumin exerts as plasma expander as well, attributed to its oncotic power. Hypoalbuminemia occurring in patients with liver fibrosis and cirrhosis endangered effective blood volume and impaired intestinal mucosal barrier, resulting in translocation of bacteria or bacterial products and local inflammation [45]. In chronically stressed mice, the reduction of Lactobacillus-derived reactive oxygen species may undermine the inhibition of indoleamine 2,3-dioxygenase and consequently increase the conversion of tryptophan to kynurenine in the intestine, which is able to traverse the blood brain barrier and linked to depression-like behavioral alterations [46]. Besides, excessive production of indole derivatives by the gut bacteria have shown neurodepressive properties [47], suggesting that microbiota dysbiosis may have an essential role in changing the functions of gut–brain axis. These may explain why the association between serum albumin and depression was significant at fibrosis stage.

Another notable discovery of our study is that there is a point of inflection and the association is much stronger when the serum albumin is higher than 3.4 g/dL among patients with LSM ≥8.2 kPa. The structural integrity of albumin is vulnerable to systemic inflammation and oxidative stress as occurs in liver fibrosis and cirrhosis [48]. Oxidative damage of the cysteine-34 residue is the most common alteration [49]. Then the concept of “effective albumin concentration” has been brought into recently [50]. The proportion of albumin with complete structure and function is much lower than the measured concentration of serum albumin. The inflected point at 3.4 g/dL may suggest that the “effective albumin” decrease and reach to a “floor effect” quite early even when the measured albumin has a nearly normal level.

It is well known that the serum albumin concentration can be influenced not only by dietary protein consumed but also by capillary leakage. On the other hand, albumin levels can decline rapidly particularly in patients with advanced fibrosis and cirrhosis because of reduced synthesis by hepatocytes. However, the confounding effects of these factors could not be controlled for the present study yet. Therefore, our results need to be interpreted with caution and further study in large population is necessary to explore whether there is significant association between serum albumin and depression in each stage of liver fibrosis.

It is noteworthy that there are some limitations in the present study. First, this study is a cross-sectional research; thus, it is unclear whether there is a causal association between serum albumin and depression among CLD patients. Second, the sample size of this study is relatively small. Although the smooth fitting curves of male and female have similar tendency, only significant association was found in female. Because of limited sample size, participants with LSM ≥8.2 kPa were not divided with advanced fibrosis and cirrhosis and we used only one threshold to define liver fibrosis although we agree with that thresholds for a certain fibrosis stage do differ with different etiologies. Hence, further studies with larger sample are needed to identify the association in different gender, different fibrosis stage and different etiologies. Third, there remains the possibility of bias caused by other confounding factors that were not included in this study.

## Conclusion

The present study suggests an inverse linear association between serum albumin and depressive symptoms in CLD patients. In particular, the inverse association may be limited among patients with CAP < 274 dB/m and patients with LSM ≥8.2 kPa, suggesting that this association was varied in different liver histological stages. This finding supports that serum albumin could be regarded as a warning marker for depressive symptoms in CLD patients, and that several strategies such as protein supplementation and dietary modification could be taken to against the risk of depression. Further prospective cohort studies are warranted to confirm the role of serum albumin in depression among CLD patients.

## Data Availability

The datasets analyzed during the present study were provided at the National Center for Health Statistics at the Centers for Disease Control. https://www.cdc.gov/nchs/nhanes/index.htm
